# The risk of atrial fibrillation in patients with non-alcoholic fatty liver disease and a high hepatic fibrosis index

**DOI:** 10.1038/s41598-020-61750-4

**Published:** 2020-03-19

**Authors:** Hyo Eun Park, Heesun Lee, Su-Yeon Choi, Hua Sun Kim, Goh Eun Chung

**Affiliations:** 1 Division of Cardiology, Department of Internal Medicine, Healthcare System Gangnam Center, Seoul National University Hospital, Seoul, Korea; 2 Division of Radiology, Department of Internal Medicine, Healthcare System Gangnam Center, Seoul National University Hospital, Seoul, Korea; 3 Division of Gastroenterology and Hepatology, Department of Internal Medicine, Healthcare System Gangnam Center, Seoul National University Hospital, Seoul, Korea

**Keywords:** Non-alcoholic fatty liver disease, Atrial fibrillation, Liver fibrosis

## Abstract

Previous epidemiological studies focusing on the association between liver disease and atrial fibrillation (AF) show interesting but inconsistent findings. Patients with liver disease have a higher AF risk; however, it is unknown whether the liver fibrosis index can predict AF risk. The medical records of a healthy population undergoing routine health examinations at Healthcare System Gangnam Center, Seoul National University Hospital, were reviewed retrospectively. After excluding subjects with a history of liver disease and known cardiovascular disease, 74,946 subjects with nonalcoholic fatty liver disease (NAFLD) were evaluated. The mean age was 51 ± 11 years, and 71.9% were male. AF was found in 380 (0.5%) subjects. Using univariate analyses, age, male sex, body mass index, hypertension, and diabetes were significantly associated with AF. The fibrosis 4 index (FIB 4) showed significant correlations with AF [unadjusted odds ratio (OR) 3.062 and 95% confidence interval (CI) 2.605–3.600, p = 0.000; adjusted OR 2.255 and 95% CI 1.744–2.915, p = 0.000, with cardiometabolic risk factors adjusted]. In conclusion, NAFLD subjects with higher FIB 4 were associated with increased AF risk. The noninvasive determination of liver fibrosis indices can have clinical implications on the early identification of NAFLD in patients at risk for AF.

## Introduction

Atrial fibrillation (AF) is gradually increasing in incidence and prevalence in Korea^1^. AF incidence increased more than 1.12-fold from 2008 to 2015, and the AF prevalence increased by 1.68-fold during the same period. An aging population and increasing comorbidities associated with the aging process have been suggested as explanations for these increases. A substantial increase in mortality and morbidity, reducing the quality of life in AF patients, is becoming a serious medical problem in Korea^1,2^. In addition to abnormal substrate and triggering ectopic foci in the heart, various inflammatory markers have been studied to identify a link between AF and systemic inflammation, but results are inconsistent^3^.

The association of AF and liver disease, with increasing prevalence of non-alcoholic fatty liver disease (NAFLD) and subsequent cirrhosis world-wide, have shown interesting results. From various previous studies regarding relation between liver diseases and AF, results have been somewhat inconsistent. A recent meta-analysis reported an approximately two-fold increased risk of AF among NAFLD patients compared with subjects without NAFLD^4^. Patients with liver cirrhosis have a 46% increased AF risk compared to controls, after covariates were adjusted^5^. In that study, the AF risk was higher in a population younger than 65 years of age, without known cardiovascular comorbidities. Another study using the fatty liver index showed an increased risk for new-onset AF in subjects with NAFLD without significant coronary disease. NAFLD predisposes individuals to AF, independent of known risk factors for atherosclerosis^6^. Shared risk factors, epicardial fat and related hormonal and cytokine activities, myocardial tissue remodeling in NAFLD have been suggested as possible explanation for linking mechanism between AF and NAFLD^7–9^.

From epidemiological studies, surrogate markers of fibrosis have been established as the best predictor of overall mortality, cardiovascular mortality and liver-related mortality in subjects with NAFLD^10^. Fibrosis indices should be calculated in all NAFLD subjects in order to determine those with a greater than medium-risk for fibrosis or advanced fibrosis according to serum fibrosis markers. These patients, independent of elevated liver enzyme levels, should be referred to a specialist. The predictive values of advanced fibrosis markers were evaluated to clarify the association between AF and liver disease^11,12^; however, the significant liver fibrosis marker levels in fatty liver disease patients, in association with AF, have not been investigated. Considering the potential risk of high levels of fibrosis biomarkers in fatty liver disease subjects, and the clinical significance of fibrosis markers in liver-related diseases, the association of AF with increased serum fibrosis indices in patients with NAFLD is of great interest.

In this study, we evaluated the association between AF and NAFLD, in terms of liver fibrosis indices, in an asymptomatic Korean population that underwent routine health evaluations.

## Methods

### Study population

This study was based on a retrospective review of medical records of all subjects who underwent routine health evaluations at the Seoul National University Hospital Gangnam Center from 2003 to 2017. Most of the study population voluntarily paid for their health check-ups, whereas others were supported by their companies. For most subjects visiting our center, blood pressure, blood tests, electrocardiogram and chest x-ray are performed routinely, as basic screening tests, for a comprehensive evaluation for medical status of each person. We identified 342,407 subjects who had an electrocardiogram in their records. Those with significant alcohol consumption (n = 52,174)^13^, positive hepatitis B antigen (n = 10,643) and positive hepatitis C virus antibody (n = 2,617) were excluded. We also excluded 27,908 subjects who lacked ultrasonography data and 10,330 subjects who did not have laboratory assessments. A total of 238,735 subjects were evaluated and fatty liver disease was found in 74,946 subjects.

Based on subject-recorded questionnaires and medications, the comorbidities of each subject were reviewed, and alcohol consumption was calculated. The study subjects were categorized as never smoker, ex-smoker and current smoker. Hypertension was defined as taking antihypertensive medications; diabetes was defined as taking any glucose-lowering agents; and hypercholesterolemia was defined as taking lipid-lowering agents. A previous history of stroke, transient ischemic attack, heart failure and other vascular disease including prior myocardial infarction or peripheral arterial disease was also reviewed.

The study protocol conformed to the ethical guidelines of the 1975 Declaration of Helsinki and was approved by the Institutional Review Board of Seoul National University Hospital (No 1801-099-917). Because the current study was performed with a retrospective design using a database and medical records, informed consent was waived by the board. All authors had access to the study data and had reviewed and approved the final manuscript.

### Measurement of anthropometric and laboratory parameters

The methods employed in this study have been described in detail elsewhere^14^. The body weight, height and waist circumference (WC) were measured on the day of the exam. Using height and body weight measured using a digital scale body mass index (BMI) was calculated according to the formula BMI = weight (kg)/height (m^2^). WC was measured by a well-trained nurse, at the midpoint between the lower costal margin and the iliac crest. All subjects were fasted for at least 12 hours prior to blood sampling; complete blood cell counts, aspartate aminotransferase (AST), alanine aminotransferase (ALT), gamma-glutamyl transpeptidase (GGT), total cholesterol, triglyceride (TG), high-density lipoprotein (HDL) cholesterol, fasting glucose, glycated hemoglobin (HbA1c), serum blood urea nitrogen and creatinine were measured. Low-density lipoprotein (LDL) cholesterol was calculated using an equation in subjects with a TG less than 400 mg/dL. In subjects with a TG of at least 400 mg/dL, the measured LDL cholesterol was used for analysis.

### Serum markers of fibrosis

To estimate the severity of fibrosis, AST to platelet ratio index (APRI), and the Fibrosis-4 score (FIB 4) were calculated. The APRI^15^ and FIB 4^16^ were calculated based on the formulas: APRI = [AST/upper limit of normal]/platelet count [10^9^/L] × 100), FIB 4 = age(years) × AST[U/L]/(platelets [10^9^/L] × (ALT[U/L]^1/2^) The cut-off values for APRI were <0.5 for low and ≥1.5 for high probability of advanced fibrosis. FIB 4 was categorized into three groups with a cut-off <1.30 for low, 1.3–2.67 for indeterminate, >2.67 for high probability of advanced fibrosis^17^.

### Electrocardiographic examination

A standard 12-lead electrocardiogram (ECG) was performed in all patients. With the patient in the supine position, 10 electrodes were placed on the limbs and chest surface, and an ECG was obtained over a duration of 10 seconds and read by three different cardiologists.

### Diagnosis and grade of NAFLD via ultrasonography

Hepatic ultrasonography (Acuson Sequoia 512; Siemens, Mountain View, CA, USA) was performed by experienced radiologists who were blinded to the clinical and laboratory data. Fatty liver was diagnosed and graded semi-quantitatvely using criteria of Saadeh *et al*.^18^ The characteristic ultrasonographic features were evident contrast between the liver and kidney, bright liver, vessel blurring and focal sparing. Using characteristic radiologic findings the severity of fatty liver was categorized as mild, moderate and severe faty liver. A slight diffuse increase in bright homogenous echoes in the liver parenchyma and the normal appearance of the diaphragm and portal and hepatic borders was defined as mild fatty liver. Moderate fatty liver was defined as a diffuse increase in bright echoes in the liver parenchyma with slightly impaired appearance of the peripheral portal and hepatic vein borders. Severe fatty liver was defined as a marked increase in bright echoes at a shallow depth with deep attenuation and impaired appearance of the diaphragm and marked vascular blurring^18^.

### Statistical analyses

In this study, fatty liver and hepatic fibrosis indices were evaluated for association with atrial fibrillation. Statistical analysis were conducted with SAS version 9.3 (SAS Institute, Inc., Cary, NC, USA). Data are expressed as the means ± standard deviation for continuous variables and as frequencies for categorical variables. We used chi-square tests for the categorical variables and Student’s t-test for continuous variables to compare the baseline characteristics between groups. Associations between AF and multiple fibrosis markers were estimated from the odds ratios (OR) and the 95% confidence intervals (CI) using multiple logistic regression. Covariates included in multivariate logistic regression models were selected as potential confounding factors based on their significance in univariate analyses. A p-value less than 0.05 was considered statistically significant.

## Result

### Baseline characteristics

Among 74,946 subjects with fatty liver, AF was diagnosed in 380 (0.5%) subjects. The mean age of all subjects was 51 ± 11 years, and 71.9% of study population was male. Male gender was more common both in total and AF populations. Table 1 shows the clinical and biochemical characteristics of participants stratified by presence or absence of AF. In subjects with AF, traditional risk factors of atherosclerosis were significantly more common: hypertension (55.6% vs 39.0% in AF versus (vs) control group respectively, p = 0.000), diabetes mellitus (29.7% vs 16.5%, in AF vs control group respectively, p = 0.000), history of smoking (68.5% vs 60.6%, in AF vs control group respectively, p = 0.000) were more common. WC (92 ± 7 vs 89 ± 8 cm in AF vs control group respectively, p = 0.000) and GGT (59 ± 66 vs 46 ± 51, in AF vs control group respectively, p = 0.000) were higher in the AF group compared to the control group.

**Table 1:** Baseline characteristics of study population in subjects with fatty liver.

Variable	Total population	Control	Afib	P-value
	(N = 74946)	(N = 74555)	(N = 380)	
Age, years	50.98 ± 10.59	50.94 ± 10.58	59.45 ± 10.11	0.000
Male sex, n(%)	53860 (71.87%)	53522 (71.79%)	334 (87.89%)	0.000
Hypertension, n(%)	19149 (39.11%)	18988 (39.01%)	155 (55.56%)	0.000
Diabetes mellitus, n(%)	7208 (16.58%)	7138 (16.51%)	69 (29.74%)	0.000
Hypercholesterolemia, n(%)	13858 (29.04%)	13768 (29.01%)	83 (33.47%)	0.141
Smoking, n(%)				0.000
Never	25618 (39.31%)	25509 (39.34%)	103 (31.50%)	
Ex-smoker	24044 (36.89%)	23866 (36.81%)	176 (53.82%)	
Current-smoker	15515 (23.80%)	15467 (23.85%)	48 (14.68%)	
BMI, kg/m^2^				0.000
<23	35559 (47.74%)	35414 (47.79%)	143 (37.63%)	
23–25	34430 (46.22%)	34215 (46.17%)	211 (55.53%)	
≥25	4498 (6.04%)	4472 (6.04%)	26 (6.84%)	
WC, cm	89.44 ± 7.59	89.43 ± 7.59	92.37 ± 7.12	0.000
***Laboratory findings***				
AST, IU/L	26.83 ± 15.96	26.83 ± 15.98	28.12 ± 11.38	0.029
ALT, IU/L	32.80 ± 22.87	32.81 ± 22.90	31.19 ± 16.79	0.064
GGT, mg/dL	46.50 ± 51.02	46.44 ± 50.93	58.97 ± 66.13	0.000
Total cholesterol, mg/dL	199.06 ± 35.46	199.13 ± 35.44	186.00 ± 36.94	0.000
Triglyceride, mg/dL	150.30 ± 92.49	150.35 ± 92.58	140.42 ± 75.09	0.011
HDL cholesterol, mg/dL	58.36 ± 15.39	58.35 ± 15.38	58.97 ± 16.75	0.479
LDL cholesterol, mg/dL	126.35 ± 36.70	126.40 ± 36.71	116.35 ± 33.82	0.000
BUN, mg/dL	14.37 ± 3.58	14.36 ± 3.57	16.13 ± 4.59	0.000
Creatinine, mg/dL	0.95 ± 0.20	0.95 ± 0.20	1.06 ± 0.21	0.000
***Fibrosis index***				
APRI^†^	0.29 ± 0.22	0.29 ± 0.22	0.34 ± 0.17	0.000
FIB 4^†^	1.08 ± 0.57	1.07 ± 0.57	1.52 ± 0.77	0.000
FIB 4‡				0.000
<1.3	56302 (75.70%)	56134 (75.87%)	168 (45.04%)	
1.3–2.67	17048 (22.92%)	16851 (22.77%)	186 (49.87%)	
>2.67	1025 (1.38%)	1006 (1.36%)	19 (5.09%)	
APRI‡				0.157
<0.5	69096 (92.90%)	68747 (92.91%)	339 (90.88%)	
≥0.5	5283 (7.10%)	5248 (7.09%)	34 (9.12%)	
***Radiology findings***				0.156
Mild fatty liver	43021 (57.40%)	42780 (57.38%)	235 (61.84%)	
Moderate fatty liver	26381 (35.20%)	26261 (35.22%)	116 (30.53%)	
Severe fatty liver	5544 (7.40%)	5514 (7.40%)	29 (7.63%)	

ALT, alanine aminotransferase; AST, aspartate aminotransferase; APRI, aspartate aminotransferase to platelet ratio index; BUN, blood urea nitrogen; BMI, body mass index; FBS, fasting blood sugar; FIB 4, Fibrosis-4 index; GGT, gamma-glutamyl transpeptidase; HbA1C, glycated hemoglobin; HDL, high-density lipoprotein; LDL, low-density lipoprotein; WC, waist circumferenece.

^†^As continuous variable.

‡As categorical variable.

APRI and FIB 4 were calculated and categorized into three groups each. Both APRI and FIB 4 were higher in subjects with AF (0.34 ± 0.17 vs 0.29 ± 0.22, p = 0.000 for APRI, 1.52 ± 0.77 vs 1.07 ± 0.57, p = 0.000 for FIB 4). The AF group had significantly more subjects with higher FIB-4 values than the control group (p = 0.000). There were no significant differences in degree of fatty liver from radiologic findings using ultrasonography (p = 0.156). Subjects with higher APRI level (≥0.5) had more AF compared to control (9.12% vs 7.09%, *P* = 0.157).

### Association of hepatic fibrosis with AF in subjects with NAFLD

The association of each parameter with AF was evaluated using univariate analyses (Table 2). Age (OR 1.079, 95% CI 1.069–1.090, p = 0.000), male gender (OR 2.853, 95% CI 2.096–3.885, p = 0.000) and greater BMI (OR 1.336, 95% CI 1.137–1.570, p = 0.000) were associated with AF. Among traditional risk factors of atherosclerosis, hypertension (OR 1.955, 95% CI 1.542–2.477, p = 0.000) and diabetes mellitus (OR 2.141, 95% CI 1.614–2.840, p = 0.000) showed significant association with AF. Hypercholesterolemia and smoking history were not significantly associated with AF (p = 0.123 for hypercholesterolemia and p = 0.758 for smoking history).

**Table 2:** Univariate analysis for association of fibrosis parameters with AF in subjects with fatty liver.

Variables	Odds ratio	95% Confidence Interval		p-value
		Lower	Upper	
Age, years	1.079	1.069	1.090	0.000
Male sex	2.853	2.096	3.885	0.000
Hypertension	1.955	1.542	2.477	0.000
Diabetes mellitus	2.141	1.614	2.840	0.000
Hypercholesterolemia	1.231	0.945	1.604	0.123
Smoking	0.978	0.851	1.125	0.758
BMI, kg/m^2^	1.336	1.137	1.570	0.000
FBS, mg/dL	1.008	1.005	1.012	0.000
HbA1C, %	1.386	1.280	1.501	0.000
Total cholesterol, mg/dL	0.989	0.986	0.992	0.000
Triglyceride, mg/dL	0.999	0.997	1.000	0.035
HDL cholesterol, mg/dL	1.003	0.996	1.009	0.440
LDL cholesterol, mg/dL	0.991	0.987	0.994	0.000
AST, IU/L	1.002	0.999	1.004	0.137
ALT, IU/L	0.996	0.991	1.002	0.170
FIB 4^†^	1.437	1.333	1.548	0.000
FIB 4^‡^	3.062	2.605	3.600	0.000
APRI^†^	1.216	1.079	1.371	0.001
APRI^§^	1.266	0.909	1.763	0.163

ALT, alanine aminotransferase; AST, aspartate aminotransferase; APRI, aspartate aminotransferase to platelet ratio index; BMI, body mass index; FBS, fasting blood sugar; FIB 4, Fibrosis-4 index; HbA1C, glycated hemoglobin; HDL, high-density lipoprotein; LDL, low-density lipoprotein.

^†^As continuous variable.

^‡^In category: low, intermediate, high risk.

^§^Low risk versus intermediate-high risk.

FIB 4 showed significant correlations with AF, both as continuous and categorical variables (OR 1.437, 95% CI 1.333–1.548, p = 0.000 as a continuous variable, OR 3.062, 95% CI 2.605–3.600, p = 0.000 as a categorical variable). APRI showed a significant correlation with AF when assessed as a continuous variable (OR 1.216, 95% CI 1.079–1.371, p = 0.001), but was not significantly correlated with AF as a categorical variable (p = 0.163).

To adjust confounding variables for AF, multivariate models were investigated as shown in Table 3. APRI and FIB 4 were evaluated as hepatic fibrosis indices. Age greater than or equal to 65 years, male sex, BMI, hypertension, diabetes mellitus, hypercholesterolemia, smoking were adjusted as covariates. FIB 4 was significant association with AF (OR 2.255, 95% CI 1.744–2.915, p = 0.000 as a categorical variable), but APRI did not show significant associations (p = 0.745).

**Table 3:** Multivariate analysis for association of fibrosis parameters with AF in subjects with fatty liver.

Variables	Odds Ratio	95% Confidence Interval		P-value
		Lower	Upper	
**Model I**				
Age≥65 years	4.505	3.255	6.235	0.000
Male sex	2.598	1.682	4.014	0.000
BMI, kg/m^2^	1.452	1.15	1.833	0.002
Hypertension	1.229	0.898	1.681	0.198
Diabetes mellitus	1.588	1.098	2.296	0.014
Hypercholesterolemia	0.928	0.644	1.339	0.690
Smoking	0.842	0.683	1.038	0.107
APRI^†^	1.081	0.676	1.731	0.745
**Model II**				
Age≥65 years	2.789	1.956	3.978	0.000
Male sex	2.363	1.529	3.654	0.000
BMI, kg/m^2^	1.457	1.156	1.837	0.001
Hypertension	1.157	0.846	1.581	0.362
Diabetes mellitus	1.499	1.037	2.166	0.031
Hypercholesterolemia	0.918	0.636	1.324	0.645
Smoking	0.869	0.702	1.075	0.196
FIB 4^‡^	2.255	1.744	2.915	0.000

APRI, aspartate aminotransferase to platelet ratio index; BMI, body mass index; FIB 4, Fibrosis-4 index.

^†^low risk versus intermediate-high risk.

^‡^in category: low, intermediate, high risk.

## Discussion

In this study, we evaluated the association between AF and NAFLD in terms of hepatic fibrosis indices in a population who underwent routine health examinations. NAFLD was associated with an increased AF risk, especially in those with higher FIB 4. Our results indicate that even after adjusting for traditional risk factors of atherosclerosis, subjects with advanced fibrosis index have an increased risk of AF.

NAFLD is significantly associated with the development of coronary artery calcium deposits^19^, the presence of vulnerable plaques in coronary arteries^20^ and increased arterial stiffness^21,22^. Furthermore, NAFLD has potential to evolve into cirrhosis and malignancy, and early identification of those at higher risk of clinically significant fibrosis has been suggested to be the main goal for management of NAFLD patients by clinical guidelines^23^.

Among the different screening options for NAFLD, the gold standard methods, liver biopsy and proton magnetic resonance spectroscopy, are either too invasive or too expensive to be used for the majority of asymptomatic individuals. Thus for screening purpose, ultrasonography or serum biomarkers have been suggested for diagnosis of NAFLD and liver fibrosis^23^. NAFLD fibrosis score and FIB 4 index are both cost-effective and highly sensitive indices to identify patients with advanced fibrosis.

For identification of NAFLD patients who are likely to have an adverse clinical outcome, surrogate fibrosis markers can be used as prognosticators of overall mortality, cardiovascular mortality and liver-related mortality^24,25^. Serum fibrosis biomarkers have demonstrated prognostic values and are thus recommended for use in all NAFLD subjects to rule out significant fibrosis^10^. The patients with high levels of liver fibrosis markers have shown significant association with poor cardiovascular outcome and should therefore be referred to specialists for evaluation of comorbidities^26,27^.

Despite slow progression rate of disease in general, NAFLD and associated pro-atherogenic milieu significantly increases other combined cardiology problems, in addition to coronary artery disease. Echocardiographic and electrocardiographic abnormalities have been reported in patients with NAFLD^28,29^. In particular, NAFLD is associated with a threefold higher risk of persistent heart block, particularly in patients with type 2 diabetes mellitus and NAFLD with advanced fibrosis (as estimated by the FIB-4 score)^22^.

Several studies have confirmed the association between AF and NAFLD. A prospective 10-year follow-up study pointed to an increased risk of incident AF in patients with NAFLD independent of other risk factors^30^. Another study showed that in hospitalized patients with type 2 diabetes NAFLD was associated with an increased persistent or permanent AF prevalence^31^. A recent longitudinal study showed that NAFLD, as assessed by fatty liver index, was independently associated with increased risk of new-onset AF^6^. However, in this study, liver steatosis was assessed by surrogate biochemical markers and not by imaging. We assessed a large group of apparently healthy individuals and is thus more likely to reflect results for the general population. Based on the ultrasonographic detection of liver steatosis, there was a significant association between AF and advanced fibrosis in NAFLD patients in our study, independent of traditional cardiac risk factors.

The mechanisms that link NAFLD, especially in advanced fibrosis patients, to AF are poorly understood. Currently, several mechanisms have been suggested to explain the higher AF risk in patients with NAFLD. An increased inflammatory burden and proatherogenic milieu have been suggested as possible mechanisms^32^. An association between NAFLD and left ventricular diastolic dysfunction was also suggested to induce AF via various mechanisms^33–35^. Unfortunately, not all study subjects have undergone echocardiography routinely in our study. A possible link between NAFLD and autonomic dysfunction may also contribute to an increased AF risk. Liver disease affects circulating inflammatory peptides and leads to autonomic dysfunction, thereby creating a proarrhythmic state and a subsequently increased AF risk^36–38^. NAFLD and advanced liver disease have been shown to be independent risk factors for autonomic dysfunction^39^; variations of sympathovagal balance seem to play a role in the initiation and perpetuation of AF^40,41^.

Although current evidence does not fully explain exact pathophysiology or mechanisms linking advanced fibrosis in NAFLD with an increased AF risk, our results show that the severity of liver fibrosis affects the AF risk, even in NAFLD patients. Advanced fibrosis in NAFLD patients is significantly correlated with coronary artery calcification and coronary artery disease, which are major risk factors for AF development^42^. Unfortunately coronary artery imaging was not performed in all study subjects for evaluation in this study.

Cardiovascular comorbidities and outcomes are traditionally underestimated in liver disease patients. Physicians tend to focus on treating renal dysfunction and liver-related morbidities and mortalities in liver fibrosis patients. Recent reports have drawn attention to the cardiovascular aspects of fatty liver disease, which are becoming the major cause of liver transplantation in westernized societies^43,44^.

In our study we have shown that severity of hepatic fibrosis reflected using biomarkers is independently associated with increased risk of AF, even after adjusting for other traditional cardiac risk factors. With the increasing prevalence of AF in progressive liver disease, even in healthy patients without definite cirrhosis, physicians should be aware of the emerging risk of AF and consider regular health evaluations for new-onset AF in NAFLD subjects and high fibrosis indices. With the increasing AF incidence, the management of complications and risk of thromboembolism using preventive or therapeutic anticoagulation are additional issues to be investigated.

### Limitation

First, the current study was designed as a retrospective observational study; thus, we cannot provide a causal relationship or prognostic significance of fibrosis biomarkers in the AF development. However, we show that there is a significant association between the levels of liver fibrosis markers and AF. In addition to correlation between AF and NAFLD, our results help stratify the risks for those who need active screening and management to improve potential adverse outcomes. Second, since our study population consisted of asymptomatic subjects without overt liver disease, we could not obtain histology results to confirm NAFLD. However, our results provide valuable findings using noninvasive and easily applicable diagnostic methods of liver fibrosis that may be more beneficial for the majority of the population undergoing screening evaluations. Third, we cannot provide data such as echocardiography results for evaluation of diastolic dysfunction or computed tomographic findings for coronary artery disease evaluations, since our study population consisted of asymptomatic self-referred healthy individuals, without known liver disease or cardiovascular disease. Further studies are needed to investigate the ultimate outcome including coronary artery disease. Fourth, considering that the study subjects were recruited from the health check-up center, since the number of subjects with AF was too small (0.5%), there may be limitations in interpreting the results. Lastly, unfortunately we do not have all data required for NAFLD fibrosis score calculation, which is useful for identifying patients at high risk of systemic complications of NAFLD.

## Conclusion

Subjects with NAFLD and advanced fibrosis indices, especially FIB 4 scores, have an increased AF risk. The noninvasive determination of liver fibrosis indices can have clinical implications in the early identification of NAFLD in patients at risk for AF. Given that the AF incidence and prevalence is steadily increasing, AF and its associated complications are of public concern. Regular screening and active management for new-onset AF, especially in subjects with NAFLD and high fibrosis indices, should be recommended.

## References

[1] Lee SR, Choi EK, Han KD, Cha MJ, Oh S (2017). Trends in the incidence and prevalence of atrial fibrillation and estimated thromboembolic risk using the CHA2DS2-VASc score in the entire Korean population. Int. J. Cardiol..

[2] Wolf PA, Abbott RD, Kannel WB (1991). Atrial fibrillation as an independent risk factor for stroke: the Framingham Study. Stroke..

[3] Zacharia E (2019). Inflammatory Biomarkers in Atrial Fibrillation. Curr. Med. Chem..

[4] Wijarnpreecha K, Boonpheng B, Thongprayoon C, Jaruvongvanich V, Ungprasert P (2017). The association between non-alcoholic fatty liver disease and atrial fibrillation: A meta-analysis. Clin. Res. Hepatol. Gastroenterol..

[5] Lee H (2017). Cirrhosis is a risk factor for atrial fibrillation: A nationwide, population-based study. Liver Int..

[6] Roh, J. H. *et al*. Association between non-alcoholic fatty liver disease and risk of new-onset atrial fibrillation in healthy adults. *Liver Int*., 10.1111/liv.14236, [Epub ahead of print] (2019). 10.1111/liv.14236 31479572

[7] Abed HS (2013). Obesity results in progressive atrial structural and electrical remodeling: implications for atrial fibrillation. Heart Rhythm..

[8] Mahajan R (2015). Electrophysiological, Electroanatomical, and Structural Remodeling of the Atria as Consequences of Sustained Obesity. J. Am. Coll. Cardiol..

[9] Ding YH (2017). Linking atrial fibrillation with non-alcoholic fatty liver disease: potential common therapeutic targets. Oncotarget..

[10] Dulai PS (2017). Increased risk of mortality by fibrosis stage in nonalcoholic fatty liver disease: Systematic review and meta-analysis. Hepatology..

[11] Markus MR (2016). Association between hepatic steatosis and serum liver enzyme levels with atrial fibrillation in the general population: The Study of Health in Pomerania (SHIP). Atherosclerosis..

[12] Karajamaki AJ (2015). Non-Alcoholic Fatty Liver Disease as a Predictor of Atrial Fibrillation in Middle-Aged Population (OPERA Study). PLoS One..

[13] Chalasani N (2012). The diagnosis and management of non-alcoholic fatty liver disease: practice guideline by the American Gastroenterological Association, American Association for the Study of Liver Diseases, and American College of Gastroenterology. Gastroenterology..

[14] Chung GE (2016). The serum vitamin D level is inversely correlated with nonalcoholic fatty liver disease. Clin. Mol. Hepatol..

[15] Wai CT (2003). A simple noninvasive index can predict both significant fibrosis and cirrhosis in patients with chronic hepatitis C. Hepatology..

[16] Vallet-Pichard A (2007). FIB-4: an inexpensive and accurate marker of fibrosis in HCV infection. comparison with liver biopsy and fibrotest. Hepatology..

[17] McPherson S, Stewart SF, Henderson E, Burt AD, Day CP (2010). Simple non-invasive fibrosis scoring systems can reliably exclude advanced fibrosis in patients with non-alcoholic fatty liver disease. Gut..

[18] Saadeh S (2002). The utility of radiological imaging in nonalcoholic fatty liver disease. Gastroenterology..

[19] Park HE (2016). Nonalcoholic Fatty Liver Disease Is Associated With Coronary Artery Calcification Development: A Longitudinal Study. J. Clin. Endocrinol. Metab..

[20] Park HE (2019). Clinical significance of hepatic steatosis according to coronary plaque morphology: assessment using controlled attenuation parameter. J. Gastroenterol..

[21] Chung GE (2015). Nonalcoholic fatty liver disease as a risk factor of arterial stiffness measured by the cardioankle vascular index. Med..

[22] Park HE (2018). Usefulness of controlled attenuation parameter for detecting increased arterial stiffness in general population. Dig. Liver Dis..

[23] European Association for the Study of the L, European Association for the Study of D, European Association for the Study of O. EASL-EASD-EASO Clinical Practice Guidelines for the management of non-alcoholic fatty liver disease. *J. Hepatol*. **64**, 1388–1402 (2016). 10.1016/j.jhep.2015.11.004 27062661

[24] Angulo P (2013). Simple noninvasive systems predict long-term outcomes of patients with nonalcoholic fatty liver disease. Gastroenterology..

[25] Unalp-Arida A, Ruhl CE (2017). Liver fibrosis scores predict liver disease mortality in the United States population. Hepatology..

[26] Targher G, Day CP, Bonora E (2010). Risk of cardiovascular disease in patients with nonalcoholic fatty liver disease. N. Engl. J. Med..

[27] Calori G (2011). Fatty liver index and mortality: the Cremona study in the 15th year of follow-up. Hepatology..

[28] Bhatia LS, Curzen NP, Calder PC, Byrne CD (2012). Non-alcoholic fatty liver disease: a new and important cardiovascular risk factor?. Eur. Heart J..

[29] Mantovani A (2017). Nonalcoholic fatty liver disease is associated with an increased risk of heart block in hospitalized patients with type 2 diabetes mellitus. PLoS One..

[30] Targher G (2013). Non-alcoholic fatty liver disease is associated with an increased incidence of atrial fibrillation in patients with type 2 diabetes. PLoS One..

[31] Targher G (2013). Non-alcoholic fatty liver disease is associated with an increased prevalence of atrial fibrillation in hospitalized patients with type 2 diabetes. Clin. Sci..

[32] Ndumele CE (2011). Hepatic steatosis, obesity, and the metabolic syndrome are independently and additively associated with increased systemic inflammation. Arterioscler. Thromb. Vasc. Biol..

[33] Petta S (2015). Epicardial fat, cardiac geometry and cardiac function in patients with non-alcoholic fatty liver disease: association with the severity of liver disease. J. Hepatol..

[34] Chung GE (2018). Nonalcoholic fatty liver disease and advanced fibrosis are associated with left ventricular diastolic dysfunction. Atherosclerosis..

[35] Nagarakanti R, Ezekowitz M (2008). Diastolic dysfunction and atrial fibrillation. J. Interv. Card. Electrophysiol..

[36] Rangari M, Sinha S, Kapoor D, Mohan JC, Sarin SK (2002). Prevalence of autonomic dysfunction in cirrhotic and noncirrhotic portal hypertension. Am. J. Gastroenterol..

[37] Voigt MD (1997). Autonomic neuropathy in extra-hepatic portal vein thrombosis: evidence for impaired autonomic reflex arc. J. Hepatol..

[38] Patel P, Dokainish H, Tsai P, Lakkis N (2010). Update on the association of inflammation and atrial fibrillation. J. Cardiovasc. Electrophysiol..

[39] Liu YC (2013). Influence of non-alcoholic fatty liver disease on autonomic changes evaluated by the time domain, frequency domain, and symbolic dynamics of heart rate variability. PLoS One..

[40] Shen MJ, Zipes DP (2014). Role of the autonomic nervous system in modulating cardiac arrhythmias. Circ. Res..

[41] Huang WA, Dunipace EA, Sorg JM, Vaseghi M (2018). Liver Disease as a Predictor of New-Onset Atrial Fibrillation. J. Am. Heart Assoc..

[42] Song DS, Chang UI, Kang SG, Song SW, Yang JM (2019). Noninvasive Serum Fibrosis Markers are Associated with Coronary Artery Calcification in Patients with Nonalcoholic Fatty Liver Disease. Gut Liver..

[43] Samji NS (2019). Liver Transplantation for Nonalcoholic Steatohepatitis: Pathophysiology of Recurrence and Clinical Challenges. Dig. Dis. Sci..

[44] Rinella ME (2015). Nonalcoholic fatty liver disease: a systematic review. JAMA..

